# Developing CuS for Predicting Aggressiveness and Prognosis in Lung Adenocarcinoma

**DOI:** 10.3390/genes14051055

**Published:** 2023-05-08

**Authors:** Honghao Liu, Haijun Che, Mengyan Zhang, Jinyue Lv, Chengjie Pu, Jiawei Wu, Yan Zhang, Yue Gu

**Affiliations:** 1 Computational Biology Research Center, School of Life Science and Technology, Harbin Institute of Technology, Harbin 150001, China; 2 College of Pharmacy, Chengdu Airport Campus, Southwest Minzu University, Chengdu 610041, China; 3 State Key Laboratory of Agrobiotechnology, School of Life Science, The Chinese University of Hong Kong, Hong Kong 999077, China; 4 College of Pathology, Qiqihar Medical University, Qiqihar 161042, China

**Keywords:** lung adenocarcinoma, cuproptosis, single cell expression, potential drugs target, prognosis marker

## Abstract

Cuproptosis is a newfound cell death form that depends on copper (Cu) ionophores to transport Cu into cancer cells. Studies on the relationship have covered most common cancer types and analyzed the links between cuproptosis-related genes (CRGs) and various aspects of tumor characteristics. In this study, we evaluated the role of cuproptosis in lung adenocarcinoma (LUAD) and constructed the cuproptosis-related score (CuS) to predict aggressiveness and prognosis in LUAD, so as to achieve precise treatment for patients. CuS had a better predictive performance than cuproptosis genes, possibly due to the synergy of SLC family genes, and patients with a high CuS had a poor prognosis. Functional enrichment analysis revealed the correlation between CuS and immune and mitochondrial pathways in multiple datasets. Furthermore, we predicted six potential drugs targeting high-CuS patients, including AZD3759, which is a targeted drug for LUAD. In conclusion, cuproptosis is involved in LUAD aggressiveness, and CuS can accurately predict the prognosis of patients. These findings provide a basis for precise treatment of patients with high CuS in LUAD.

## 1. Introduction

Lung cancer is a prevalent and aggressive malignancy, with most patients presenting with locally advanced or metastatic disease [1]. Among the various subtypes of lung cancer, lung adenocarcinoma (LUAD) is the most common and invasive [2]. Although surgical resection, radiotherapy, chemotherapy, and immunotherapy are standard treatments for LUAD, patients often develop resistance to therapy, leading to unsatisfactory survival rates, with only 18% of patients surviving five years after diagnosis [3,4,5].

Recent research has identified a variety of mechanisms involved in cell death [6,7,8]. In addition to regulating cell death, these molecular mechanisms contribute to normal physiological functions, as well as causing pathological changes. As an essential cofactor of enzymes, copper (Cu) homeostasis is emerging as crucial in metabolic disorders and cancers [9,10,11]. Copper toxicity is accompanied by an increase in copper accumulation, which is responsible for cell death [12]. However, the molecular mechanisms underlying copper-induced cell death remain unknown. On 17 March 2022, Tsvetkov et al. first proposed the cuproptosis, a new mechanism of cell death [13]. This process is similar to the dysregulation of copper homeostasis and can be regulated by specific genes such as Ferredoxin 1 (*FDX1*). Cuproptosis is related to mitochondrial-mediated copper aggregation, protein lipolysis, and proteotoxic stress [13].

Furthermore, Cu plays a significant role in tumor growth, progression, metastasis, angiogenesis, and immune evasion. Therefore, cuproptosis shows great potential in cancer treatment [14,15,16]. However, lacking target genes for cancer and challenges associated with identifying sensitive patients have limited the clinical utility of this approach [17]. To address these obstacles, bioinformatics analysis offers theoretical guidance for exploring the regulation effects of cuproptosis, developing targeting drugs, and identifying sensitive patients from cohorts.

In this study, we used WGCNA and lightGBM algorithms to screen for prognosis-related genes associated with cuproptosis. Using these genes, we developed a cuproptosis-related score (CuS) model, which was evaluated for its prognostic value in LUAD. Additionally, function enrichment and immune cohort analyses demonstrated the association between CuS and tumor immune infiltration, as well as progression-free survival time in LUAD patients. Finally, potential therapies for patients with high CuS were identified through drug prediction.

## 2. Materials and Methods

### 2.1. Data Source

The bulk RNA-seq data were downloaded from The Cancer Genome Atlas (TCGA, https://portal.gdc.cancer.gov/, accessed on 10 May 2022) and the Gene Expression Omnibus (GEO, https://www.ncbi.nlm.nih.gov/geo/, accessed on 5 October 2022) databases. There were 572 transcriptome samples and 616 mutation samples in TCGA cohorts, 196 samples in the GSE37745 validating cohorts, 176 samples in the GSE42127 validating cohorts, 127 samples in the GSE50081 validating cohorts, and 27 samples in the GSE135222 immunotherapy cohorts.

The single-cell RNA-seq data (131669 cells, 12 samples) was downloaded from Code Ocean (https://codeocean.com/capsule/8321305/tree/v1, accessed on 15 May 2022).

### 2.2. Bulk Transcriptome Analysis

For TCGA lung adenocarcinoma transcriptome data, we filtered the samples without survival and clinical traits, and the ENSEMBL ID without annotation. The maximum value of the gene corresponding to multiple ENSEMBL IDs was taken as the gene expression value, and genes not expressed in all samples were filtered. We utilized the Wilcoxon test to compare groups and identified differentially expressed genes (DEGs) with |log2FC| > 2 and false discovery rate (FDR) < 0.05.

Subsequently, we merged DEGs with cuproptosis genes (7079 genes, Tables S1 and S2) and used weighted gene co-expression network analysis (WGCNA) [13,18] to identify modules related to prognosis. Among them, the cutoff height of the outlier samples was set to 6000, the scale-free fitting index was set to 0.9, the soft threshold power β was set to 2, the correlation type was set to “pearson”, the mergeCutHeight was set to 0.25, the minModuleSize was set to 30, and the network and TOM type was set to “unsigned”.

### 2.3. Single-Cell Transcriptome Analysis

The “Seurat” R package (version 4.2) was used to read single-cell data and merge all samples [19,20]. The number of counts (1000–100,000) and features (500–10,000), as well as the percent of mitochondrial genes (<30%), were used to filter out high-quality cells.

We visualized the top 15 principal components using UMAP, and three main cell types (immune, epithelial, and stromal) were identified using cluster marker genes. The PCA and UMAP were reused on the main cell types, and the cell type markers were adapted from Haberman et al. [21]. In the further analysis, EPCAM and PTPRC high-expression clusters were considered as contaminants and removed. For epithelial cells of tumor samples, “InferCNV” was used to identify whether the cells were malignant [22]. Compared with normal cells, epithelial clusters with abnormal copy numbers were annotated as tumor cells.

### 2.4. Cuproptosis-Related Scoring Modeling Construction

We used the two modules (including cuproptosis genes) obtained by “WGCNA” to extract the expression matrix of epithelial cells in the single-cell data. Next, we took the results of “InferCNV” as labels, and used “lightGBM” [23] to divide the expression matrix into training data, test data, and verification data (7:2:1). We set the parameter num_leaves as 15, trained classification model, and screened features. Finally, the features were used in the “AddModuleScore” function to construct the cuproptosis-related score.

### 2.5. Survival and Enrichment Analysis

The mean value was used as the grouping standard of cuproptosis-related score and cuproptosis gene expression. According to the overall survival analysis, the Kaplan–Meier curve was used to compare the survival rate difference between the high and low groups. Wilcoxon testing was used to analyze the difference between single-cell and bulk transcriptome for tumor samples with high and low cuproptosis-related scores. Enrichment analysis was conducted using differential expression genes with FDR < 0.05 and |log2FC| > 0.15 in single-cell data [24,25] and FDR < 0.05 and |log2FC| > 2 in transcriptome data. “ClusterProfiler” [26] was used to conduct KEGG and GO term gene set enrichment analyses (GSEA).

### 2.6. Immune Analysis and Drug Prediction

To explore the disparities in immune infiltration between the high and low cuproptosis-related score groups, we utilized “ESTIMATE” to estimate tumor purity, stromal score, and immune score. The drug sensitivity data of human cancer cell line were obtained from Genomics of Drug Sensitivity in Cancer (GDSC, https://www.cancerrxgene.org/, accessed on 25 October 2022), and “oncoPredict” [27] was used for drug prediction (FDR < 0.05, log2FC > 0.35) [28].

### 2.7. Statistical Analysis

The statistical analyses were performed using R (version 4.2.0). We utilized the Wilcoxon test for group comparisons, and calculated the correlation coefficient using Pearson. The data visualizations were created by the R packages “ggplot2” [29] and “ComplexHeatmap” [30]. We considered *p* < 0.05 as statistically significant and annotated the level of significance as follows: NS for no statistical significance, * for *p* < 0.05, ** for *p* < 0.01, and *** for *p* < 0.001.

## 3. Results

### 3.1. Overview

**Figure 1:**
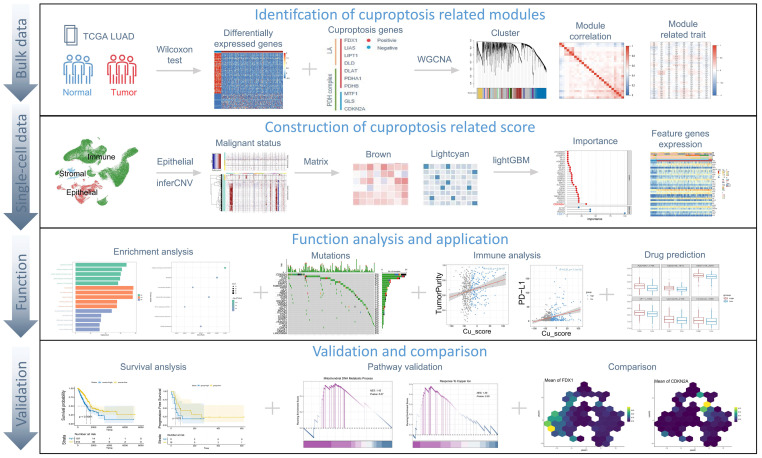
Overview of the study.

The study flowchart is presented in Figure 1.

### 3.2. Identification of Cuproptosis-Related Prognosis Modules

**Figure 2:**
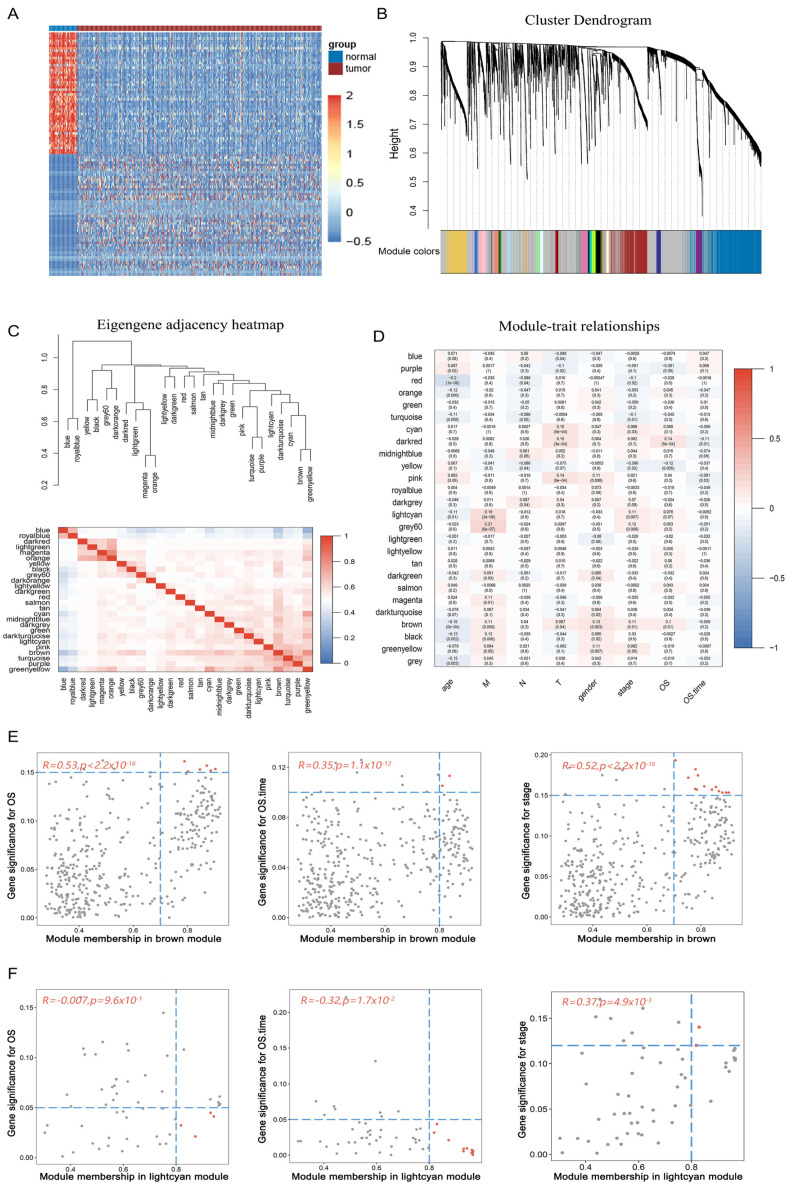
Cuproptosis genes-related modules. (**A**) Heatmap of DEGs in the TCGA lung adenocarcinoma dataset. (**B**) Clustering gene dendrograms with modules colored according to their topological overlap. (**C**) The cluster dendrogram of 26 modules with the adjacency matrix. (**D**) Correlation heatmap between modules and clinical traits. T, tumor; N, lymph node; M, metastasis. (**E**) Relationship map between MM and GS of clinical traits in brown module. (**F**) Relationship map between MM and GS of clinical traits in lightcyan module.

We downloaded transcriptome and clinical traits data of LUAD from TCGA, including 513 tumor samples and 59 normal samples. After the transcriptome samples were normalized (TPM), 7070 DEGs were identified by Wilcoxon testing (Table S1), including 6084 up-regulated genes and 986 down-regulated genes (FDR < 0.05, |log2FC| > 2). Figure 2A and Figure S1A show the differential gene expression and distribution. WGCNA analysis was performed on the DEGs and cuproptosis genes [13] (10 genes, Table S2). We filtered out obvious abnormal samples by clustering the samples with a height cutoff value of 6000, leaving 570 samples for analysis. The soft threshold power was calculated according to the scale independence and average connectivity β (Figure S1B), the gene network was constructed, and the modules were identified (the grey module genes are not clustered). Finally, 26 gene co-expression modules (Figure 2B) were constructed, of which 3 modules (brown, lightcyan and turquoise) contained cuproptosis genes (Table S2).

We calculated eigengenes for modules and clustered them based on their correlations (Figure 2C). The results showed that 26 modules can be grouped into two categories. The three modules (turquoise, brown and lightcyan) containing the cuproptosis genes were relatively close, and two of them (brown and lightcyan) have strong overall correlation with other modules; thus, they were candidate modules. Modules were correlated with clinical traits, and significant correlations were identified. As shown in Figure 2D, brown and lightcyan modules were associated with clinical stage, metastasis, and survival time. Figure 2E,F showed the significant correlation between module membership (MM) and gene significance (GS) of brown and lightcyan modules. In the brown module, the correlation between MM and GS was positive and significant in terms of survival status, survival time, and clinical staging. In the lightcyan module, the MM and GS of survival status and survival time are negatively correlated, whereas with clinical staging they are positively correlated. Among them, the survival time and clinical staging of MM and GS were significant.

### 3.3. Screening of Cuproptosis-Related Feature Genes

**Figure 3:**
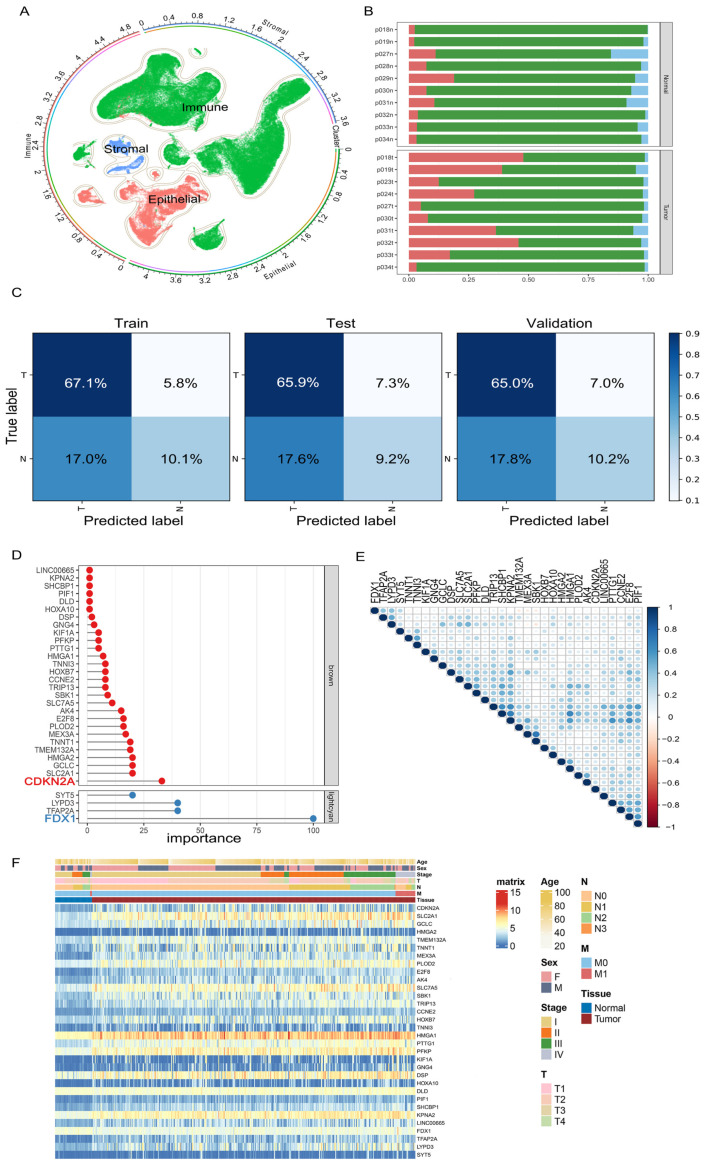
Identification of feature genes. (**A**) The UMAP of lung adenocarcinoma. (**B**) The proportion of various cell types in the samples. (**C**) The confusion matrix of the “fuse model” in training, testing, and validation data. (**D**) Feature genes and importance. (**E**) Correlation map of feature genes. (**F**) The heatmap of feature genes in TCGA cohort.

We preprocessed the downloaded single-cell data, and 114,489 high-quality cells remained after quality control and filtering. We normalized the filtered data and performed unsupervised dimensionality reduction using PCA and UMAP visualization (Figure 3A). According to the previous research [21], we annotated the main cell types (Figure S2A), and obtained 20,450 epithelial cells, 89,766 immune cells, and 4273 stromal cells, respectively. We observed the cell proportion distribution of normal and tumor samples, and found that epithelial cells accounted for a high proportion in tumor samples (Figure 3B). Combined with the clinical traits, it was found that the epithelial cell production was also different among different subtypes of LUAD. Therefore, the malignant status of epithelial cells has the potential to be used as a label for more accurate tumor classification models.

The epithelial transcriptome data were analyzed by “inferCNV”, and the corresponding barcode of the malignant cell was obtained. We extracted the single-cell transcriptome data of epithelial cells according to the gene sets of brown and lightcyan modules, respectively, and used the cell malignant status as a label. We used “lightGBM” to train binary models, and the data sets were split based on a random ratio of 7:2:1 for training, validation, and testing. The AUC of the brown module training set, test set, and verification set was 0.74 (Figure S2B); the AUC of the lightcyan module training set, test set, and verification set were 0.66, 0.65, and 0.64 (Figure S2C). The “fuse model” composed of both modules showed better performance, with an AUC of 0.82, 0.78, and 0.78 (Figure S2D). The confusion matrix indicated that the combined model had a high accuracy rate in disease prediction (Figure 3C). Based on the “lightGBM” algorithm, the brown module screened 28 feature genes, and the lightcyan module screened 4 feature genes. The cuproptosis genes Cyclin Dependent Kinase Inhibitor 2A (*CDKN2A*) and *FDX1* were the most important genes of brown and lightcyan modules, respectively (Figure 3D).

The heatmap in Figure 3F displays the expression levels of 32 featured genes in TCGA data. Some genes were differentially expressed not only between normal and tumor samples, but also in tumor samples (*CDKN2A*; Solute Carrier Family 2 Member 1, *SLC2A1*). Some genes were more different in tumor samples (*FDX1*; Dihydrolipoamide Dehydrogenase, *DLD*). We observed the correlation of the expression of these feature genes in tumor samples, and found that most of them showed significant positive correlation except *FDX1* (Figure 3E). *FDX1* was positively correlated with *DLD* expression and negatively correlated with *CDKN2A*, which was consistent with previous reports [13]. Finally, we enriched these 32 feature genes with KEGG, and the gene set was significantly enriched in carbon metabolism, T cell infection, and cell circulation pathways in cancer (Table S3).

### 3.4. Construction of Cuproptosis-Related Score

**Figure 4:**
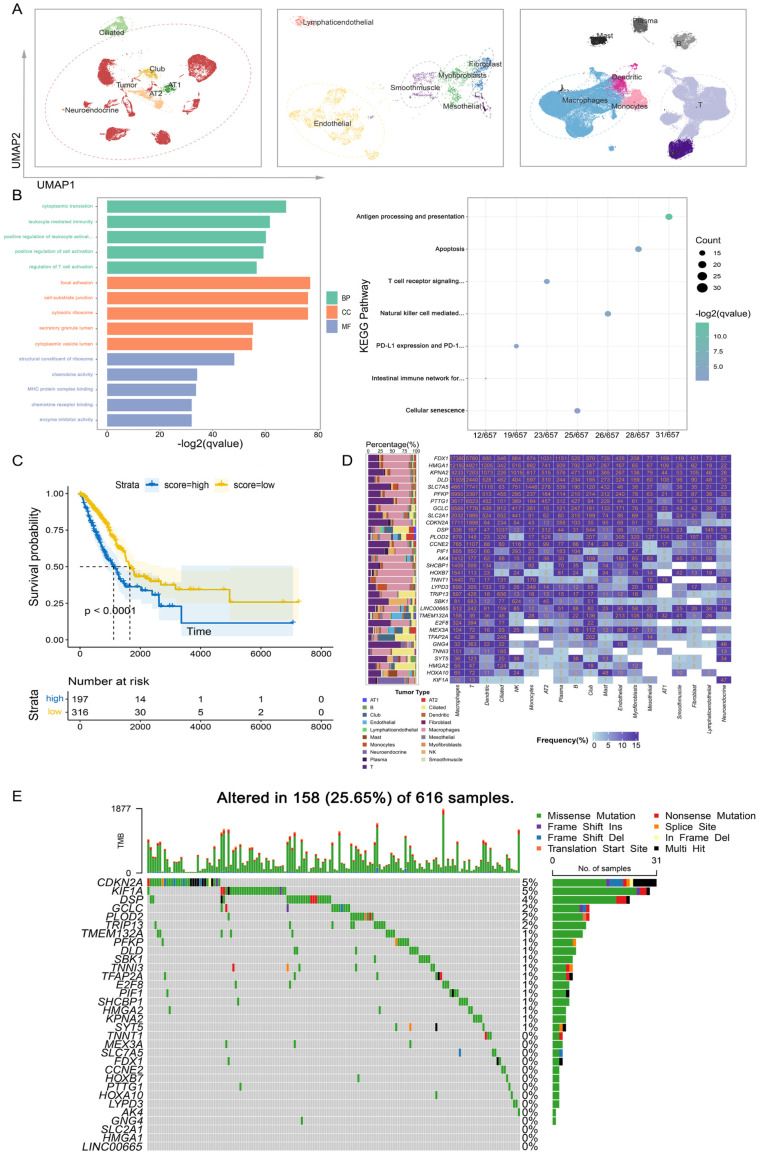
Function enrichment analysis between high and low CuS groups. (**A**) The UMAP of epithelial, immune, and stromal cells. (**B**) GO (left) and KEGG (right) analysis between high and low CuS groups in single-cell data. (**C**) Kaplan–Meier survival analysis of high and low CuS groups in TCGA data. (**D**) The frequency of feature genes in different cell types. (**E**) The somatic mutations of feature genes in TCGA samples.

The three main cell types (epithelial, immune and stromal) were classified in detail according to their corresponding marker genes (Figure 4A and Figure S3). The “AddModuleScore” function was used to calculate the cuproptosis-related score (CuS) for each cell. Between normal and tumor samples, we found that cuproptosis-related scores were significantly different in immune cells (T, Macrophages and B) and stromal cells (Endophyllal, Fibroblast and Myofibroblast) (Figure S4A). In the normal samples, the CuS was almost below the average level (Figure S4C). Among them, the scores of Lymphaticendophyllia, Mesothelial and Neuroendocrine cells were higher, and the scores of other cell types were lower (Figure S5A). In the tumor samples, according to CuS, they could be divided into high and low groups (Figure S4B), and the scores of immune cells and stromal cells were higher, whereas the scores of epithelial cells were lower (Figure S5B). We used the “FindMarkers” function to analyze the difference between high and low subtypes in tumor samples, enriched GO and KEGG for the obtained differential genes, and found that these genes were mainly enriched in immune and cell cycle-related pathways (Figure 4B). Figure 4D illustrates the expression frequency of feature genes in different cell types, indicating that a majority of these genes are expressed in immune cells (T and NK) and epithelial cells (Ciliated). This finding suggests that these genes are significantly associated with cancer occurrence and immunity.

For TCGA bulk transcriptome data, the principle of “AddModuleScore” was also used to calculate CuSs for tumor samples. According to the CuS, the transcriptome samples were divided into high and low subtypes. The Wilcoxon test was used for differential analysis, and the obtained genes were used for GO and KEGG enrichment analysis. The enriched pathways are shown in Figure S6C, mainly involving cell cycle and cell cycle-related pathways. Finally, Kaplan–Meier curves were drawn to compare the survival rate difference between high and low subtypes in tumor samples. It was found that the prognoses of samples with low CuSs was significantly better than samples with high CuSs (Figure 4C). Figure 4E displays the somatic mutations of feature genes in TCGA samples, revealing that *CDKN2A* had the highest mutation frequency. Other genes that were highly mutated include Desmoplakin (*DSP*), which can regulate immune responses; Thyroid hormone receptor interactor 13 (*TRIP13*) and E2F Transcription Factor 8 (*E2F8*) which can regulate cell cycle progression and control cell division; Glutamate-Cysteine Ligase Catalytic Subunit (*GCLC*), which affects the metabolism of non-small cell lung cancer cells; Procollagen-Lysine,2-Oxoglutarate 5-Dioxygenase 2 (*PLOD2*), which has redox activity and regulates metabolism; and Phosphofructokinase, Platelet (*PFKP*), which can promote the proliferation of tumor cells.

### 3.5. Cuproptosis-Related Score Associates with Immune Infiltration

**Figure 5:**
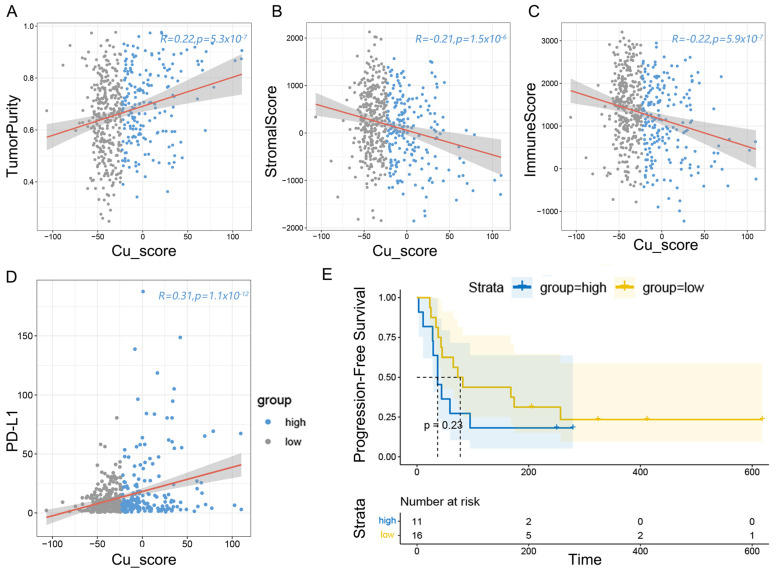
Immune characteristics of CuS in LUAD. (**A**) The CuS was positively associated with tumor purity. (**B**) The CuS was negatively associated with stromal scores. (**C**) The CuS was negatively associated with immune scores. (**D**) The CuS was positively associated with PD-L1 expression. (**E**) Progression-free survival curve in GSE135222 immunotherapy cohort.

Considering the association between CuS and immunity, we further investigated the difference in immune infiltration between subtypes with high and low CuSs. “ESTIMATE” was used to calculate tumor purity, stromal score, and immune score (Table S4). We found that the CuS in TCGA was positively correlated with the tumor purity and PD-L1 expression (Figure 5A,D), and negatively correlated with the stromal score and immune score (Figure 5B,C). We verified and got consistent results in GSE42127 (Table S4 and Figure S7). These results suggest that patients with high CuSs have a worse prognosis and are less responsive to immunotherapy. The prognosis analysis of immunotherapy revealed the same result in GSE135222 (Figure 5E).

### 3.6. Association of Mitochondrial Pathway and Drug Prediction Based on CuS

**Figure 6:**
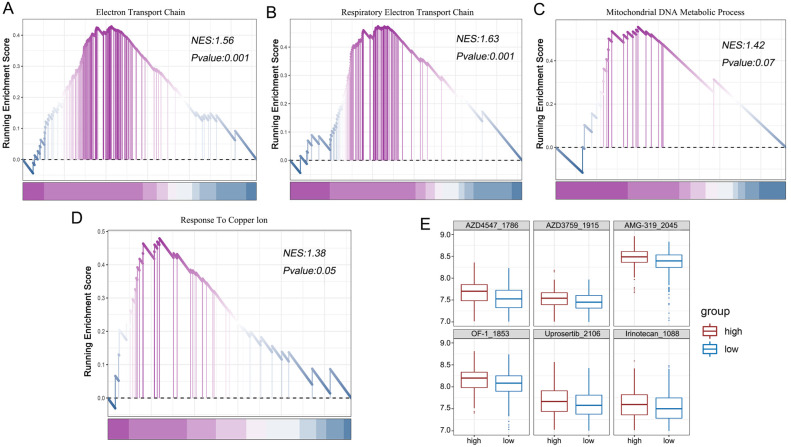
Enrichment analysis and drug prediction. The association between mitochondrial- (**A**–**C**) and copper- (**D**) related pathways and CuS in TCGA. (**E**) The potential drugs of patients with high CuSs in TCGA.

Since cuproptosis involves copper and mitochondrial-related pathways [13], we further studied the association between pathways and CuS. The pathways involved electron transport chain (GOBP_ELECTRON_TRANSPORT_CHAIN, GOBP_RESPIRATORY_ELECTRON_TRANSPORT_CHAIN), mitochondrial metabolism (GOBP_MITOCHONDRIAL_DNA_METABOLIC_PROCESS), and copper ion response (GOBP_RESPONSE_TO_COPER_ION). It was found that these pathways were positively correlated with CuSs in LUAD from TCGA cohort (Figure 6A–D). These results showed that the CuS could reveal the known biological characteristics of cuproptosis.

Due to the fact that the prognoses of patients with high CuSs is worse, we further explored the therapeutic effect of drug treatment on patients with high CuSs. According to the gene expression and drug sensitivity in the GDSC available as training data, we used “oncoPredict” to predict the potential drug of patients with high CuSs in TCGA (Table S5). This analysis produced six potential drugs, AZD4547, AZD3759, AMG-319, OF-1, Uprosertib, and Irinotecan (Figure 6E), which had the most significant differences between high and low CuS patients. Among them, AZD3759 was a targeted drug for LUAD, and the other drugs had certain curative effects on cancers. These drugs mainly functioned on DNA replication and phosphorylation kinase pathways [31,32,33,34,35,36], and may inhibit cancer by controlling cell replication.

### 3.7. Comparison between Cuproptosis Genes and Cuproptosis-Related Score

**Figure 7:**
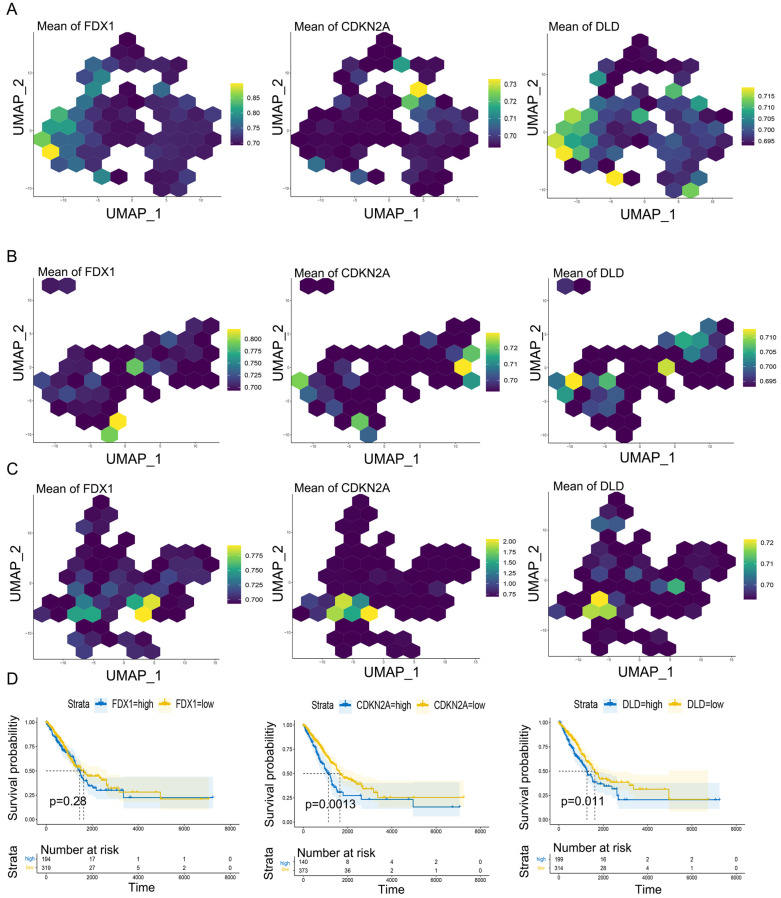
The distribution and prognosis evaluation of cuproptosis genes in various cell types. (**A**) The distribution of *FDX1*, *CDKN2A*, *DLD* in immune cells. (**B**) The distribution of *FDX1*, *CDKN2A*, *DLD* in stromal cells. (**C**) The distribution of *FDX1*, *CDKN2A*, *DLD* in epithelial cells. (**D**) The prognostic of *FDX1*, *CDKN2A*, *DLD* in TCGA cohort.

We observed the expression of cuproptosis genes (*FDX1*, *CDKN2A*, *DLD*) in various cell types: In immune cells, *FDX1* was mainly expressed in Macrophages and T cells, and *DLD* and *CDKN2A* were also expressed in B, NK and other cells (Figure 7A). In stromal cells, *FDX1* and *DLD* were highly expressed in all kinds of cells, and *CDKN2A* was mainly expressed in Mesothelial (Figure 7B). In epithelial cells, *FDX1* and *DLD* were highly expressed in all kinds of cells, and *CDKN2A* was mainly expressed in tumor (Figure 7C). These results were similar to the distribution of CuS.

We further compared the prognostic difference between cuproptosis genes (*FDX1*, *CDKN2A*, *DLD*) and CuSs in the transcriptome data. The prognosis was better when *FDX1*, *DLD*, and *CDKN2A* were low, as expressed in TCGA cohort. The correlation of *DLD*, *CDKN2A* and prognoses were significant (Figure 7D). Similarly, patients with low CuS had significantly better prognoses (Figure S8E). In the GSE37745 and GSE50081, the cuproptosis genes (*FDX1*, *CDKN2A*, *DLD*) could not well evaluate the prognosis (Figure S8A,C,D). In GSE42127, the prognosis of *FDX1* overexpression was significantly better (Figure S8B,D). These results indicated that the CuS can better evaluate the prognoses of patients than using the cuproptosis genes directly. This may be due to the synergistic effect of SLC family genes and other genes in cuproptosis [13,37,38,39].

## 4. Discussion

In most studies on cuproptosis, the screening of feature genes was only based on cuproptosis genes [40], which limited the model performance and biological significance. The occurrence of cuproptosis is not a single gene-encoded trait, and thus requires the synergistic effect of the SLC family [13,37,38,39] and other genes. We used WGCNA to divide the cuproptosis genes and the differential genes in the lung adenocarcinoma transcriptome into 26 co-expression gene modules. Finally, the co-expressed modules containing cuproptosis genes related to prognosis were extracted as candidate feature gene sets.

In recent years, the feature genes of the scoring model have been screened based on transcriptome data using cox and lasso regression methods [41,42,43,44,45]. The development of single-cell RNA sequencing technologies has made vast amounts of single-cell RNA sequencing data available [46,47,48,49,50], enabling the construction of gene networks in single cells. However, there is still no research on the use of single-cell data to build classification models for feature screening. This may be because conventional models such as cox and lasso regression are not applicable to single-cell data, and the Wilcoxon test and other methods used for single-cell data cannot be used for specific gene sets. To overcome these challenges, we used lightGBM to build a binary classification model for single-cell data and screen out feature genes. This algorithm was suitable for the characteristics of single-cell data and can effectively avoid overfitting. We used the epithelial malignant status as input labels, which has the potential to be used as a more accurate classification label for screening biomarkers.

Accordingly, we constructed the cuproptosis-related score based on the feature genes. The CuS below the average level was almost normal samples in bulk and single-cell transcriptome data. Functional enrichment analysis comparing high and low subtypes in tumor samples revealed significant involvement of immune and cell cycle-related pathways. We therefore investigated the correlation between CuS and immune infiltration. Our analysis revealed a positive correlation between CuS and tumor purity and PD-L1 expression, as well as a negative correlation with stromal score and immune score in multiple datasets. Survival analysis has shown that patients with low CuSs had significantly better prognoses. This was further confirmed by progression-free survival analysis in GSE135222. Taken together, our results show that patients with high CuS levels in LUAD have a more aggressive disease and a poorer prognosis.

In the next step, we verified the association between CuS and copper and mitochondrial-related pathways, and found that CuS conforms to the known biological characteristics of cuproptosis. Considering the poor prognosis of patients with high CuSs, we predicted six potential drugs for patients with high CuSs. These drugs involve DNA replication and phosphorylation kinase pathways, and may inhibit cancer by targeting these pathways, which is consistent with the results of the enrichment analysis. Moreover, we observed the distribution and prognosis evaluation of cuproptosis genes (*FDX1*, etc.) involved in this study. We found that prognosis evaluation of CuS swas more stable than that of cuproptosis genes. As previously mentioned, the CuS contains the SLC family [13,37,38,39] and other genes, which may function with cuproptosis genes. This also explains the lack of stability in prognostic evaluation in previous studies [40,51,52].

Despite the significant findings and contributions of our study, there were some limitations that should be addressed. A notable limitation was the absence of immunotherapy cohorts for LUAD, which prevented us from validating the immune correlation analysis of the CuS model. Moreover, the efficacy and safety of the targeting drugs in real-world settings need to be evaluated in clinical trials. Therefore, further large-scale immune validation studies and clinical research are warranted to better understand the potential applications of our findings.

## 5. Conclusions

In conclusion, our study developed the CuS using the cuproptosis-related module genes to predict the prognosis and clinical characteristics of LUAD patients. Our scoring system uncovered the association between LUAD and mitochondrial-related pathways and the immune microenvironment, and demonstrated the robustness of the CuS across multiple datasets. Furthermore, we demonstrated that cuproptosis-related module genes outperform individual cuproptosis genes in distinguishing cuproptosis-related LUAD subtypes, possibly due to the inclusion of SLC family genes [13,37,38,39]. Our identification of potential drugs targeting a high CuS provides new therapeutic options for LUAD patients. However, we acknowledge the limitations of our study, including the lack of immunotherapy cohorts for LUAD and the need for further investigation of the real-world roles of predicted drugs. Ultimately, we propose that CuS can shed light on the role of cuproptosis in LUAD and serve as a novel tool for personalized treatment.

## Data Availability

Data involved in this study can be found in the Supplementary Materials. The code involved in this study has been deposited in github: [https://github.com/Hit-Master/Cuproptosis (accessed on 30 December 2022)].

## Supplementary Materials

The following supporting information can be downloaded at: https://www.mdpi.com/article/10.3390/genes14051055/s1. Figure S1: Bulk data analysis; Figure S2: Details about the CuS model construction; Figure S3: Dotplot for annotation; Figure S4: The CuS difference in samples; Figure S5: The CuS in various cells; Figure S6: Function enrichment in TCGA; Figure S7: Immune characteristics of the CuS in GSE42127; Figure S8: Comparison between cuproptosis genes and CuS in validation datasets; Table S1: Differential Genes; Table S2: Module information; Table S3: 32 feature genes enriched in KEGG; Table S4: Estimate Score; Table S5: Drug prediction information.
