# Supplementary material for: Developing CuS for Predicting Aggressiveness and Prognosis in Lung Adenocarcinoma
Source: Genes (Basel). 2023 May 8;14(5):1055. doi: 10.3390/genes14051055 (PMC10218358; doi:10.3390/genes14051055)
Supplement: Supplementary file 1 [file genes-14-01055-s001.zip › Supplementary Figures.pdf]

Supplementary Figures

# Index

Index ........................................ 1

Fig. S1 Bulk data analysis ........................................ 2

Fig. S2 Details about the CuS model construction ........................................ 3

Fig. S3 Dotplot for annotation ........................................ 4

Fig. S4 The CuS difference in samples ........................................ 5

Fig. S5 The CuS in various cells ........................................ 6

Fig. S6 Function enrichment in TCGA........................................ 7

Fig. S7 Immune characteristics of the CuS in GSE42127 ........................................ 8

Fig. S8 Comparison between cuproptosis genes and CuS in validation datasets .... 9

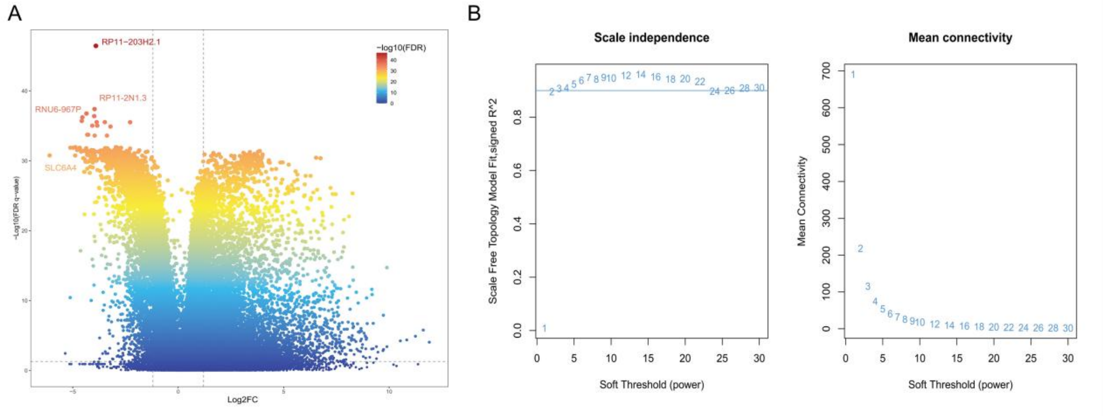


**Figure S1** Bulk data analysis. **(A)** The distribution of DEGs in Volcano plot. **(B)** Network topology analysis of soft threshold power. The left panel shows a function of scale-free fitting index (y-axis) and the soft threshold power (x-axis). The right panel shows a function of average connectivity (y-axis) and soft threshold power (x-axis).

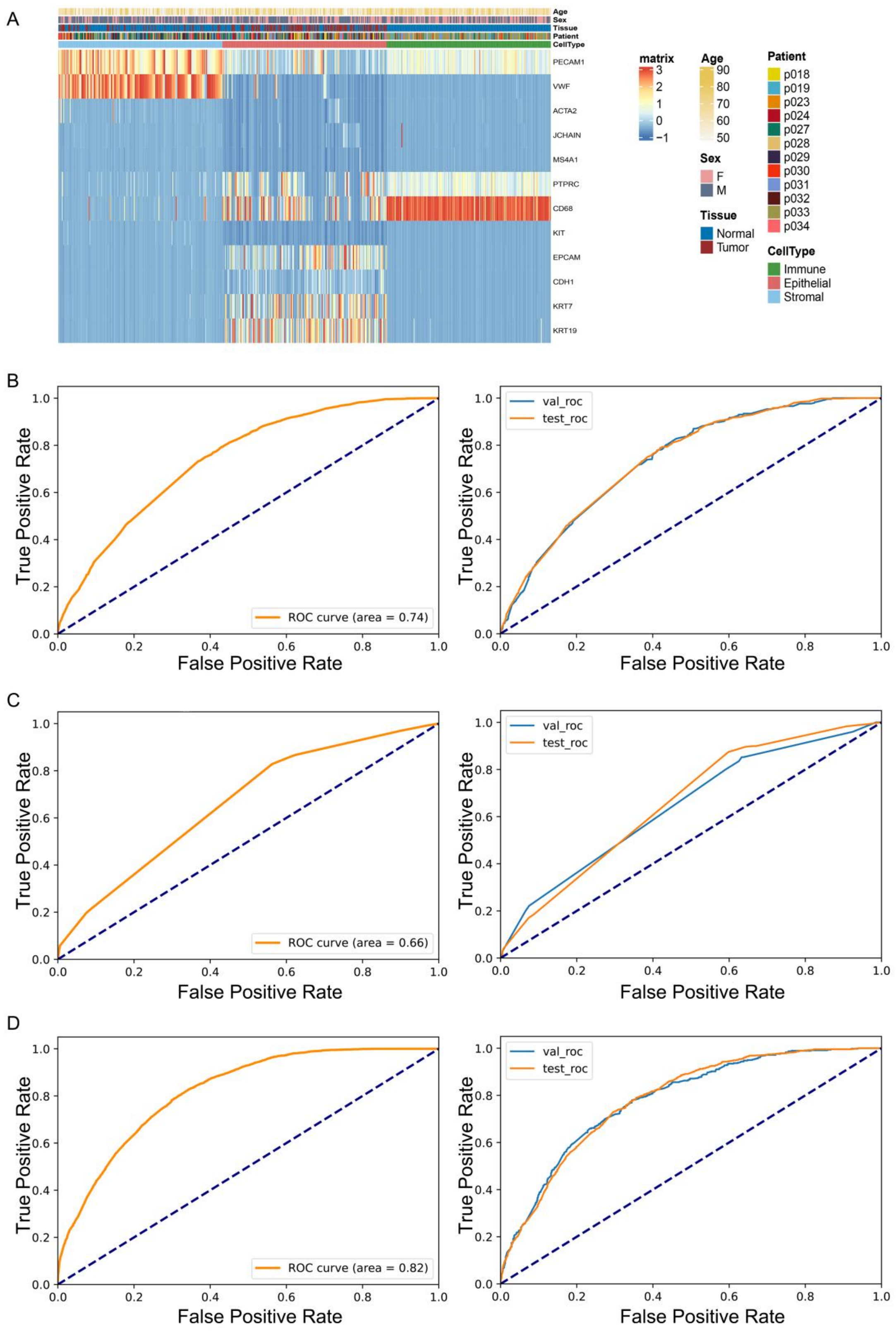


**Figure S2** Details about the CuS model construction. **(A)** Heatmap of the normalized marker expression in the main cell types. **(B)** The ROC curve of train, test and validation datasets using the brown module. **(C)** The ROC curve of train, test and validation datasets using the lightcyan module. **(D)** The ROC curve of "fuse model" in train, test and validation datasets.

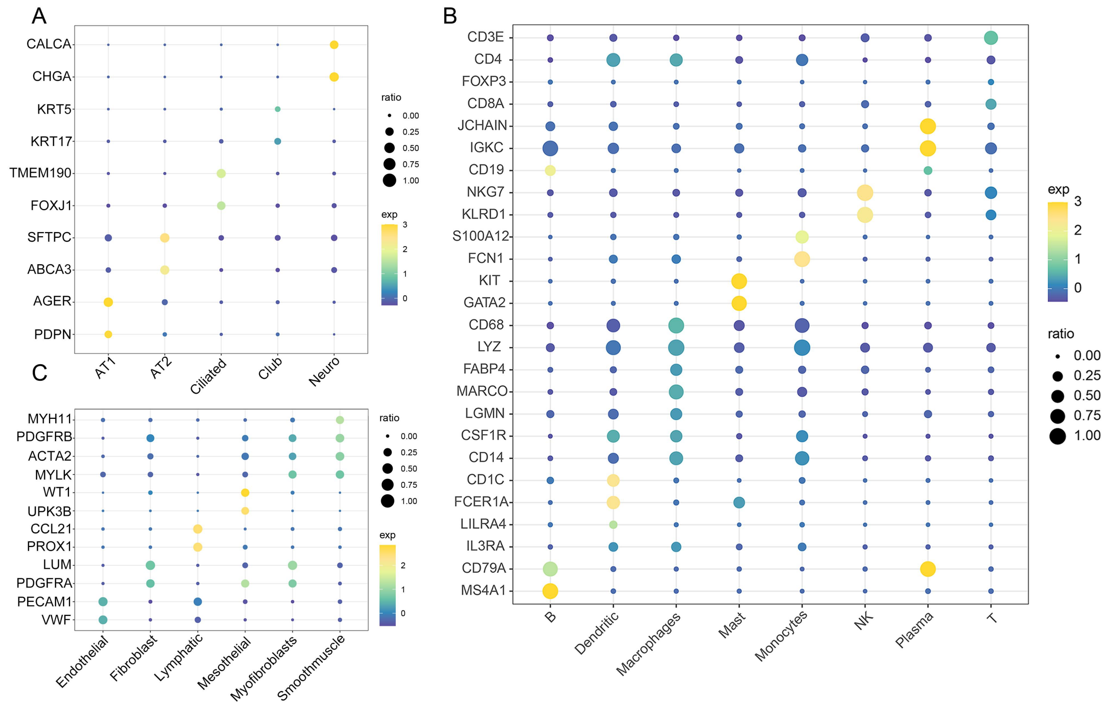


**Figure S3** Dotplot for annotation. Dotplot with marker genes used to annotate epithelial**(A)**, immune**(B)** and stromal**(C)** cell subtypes.

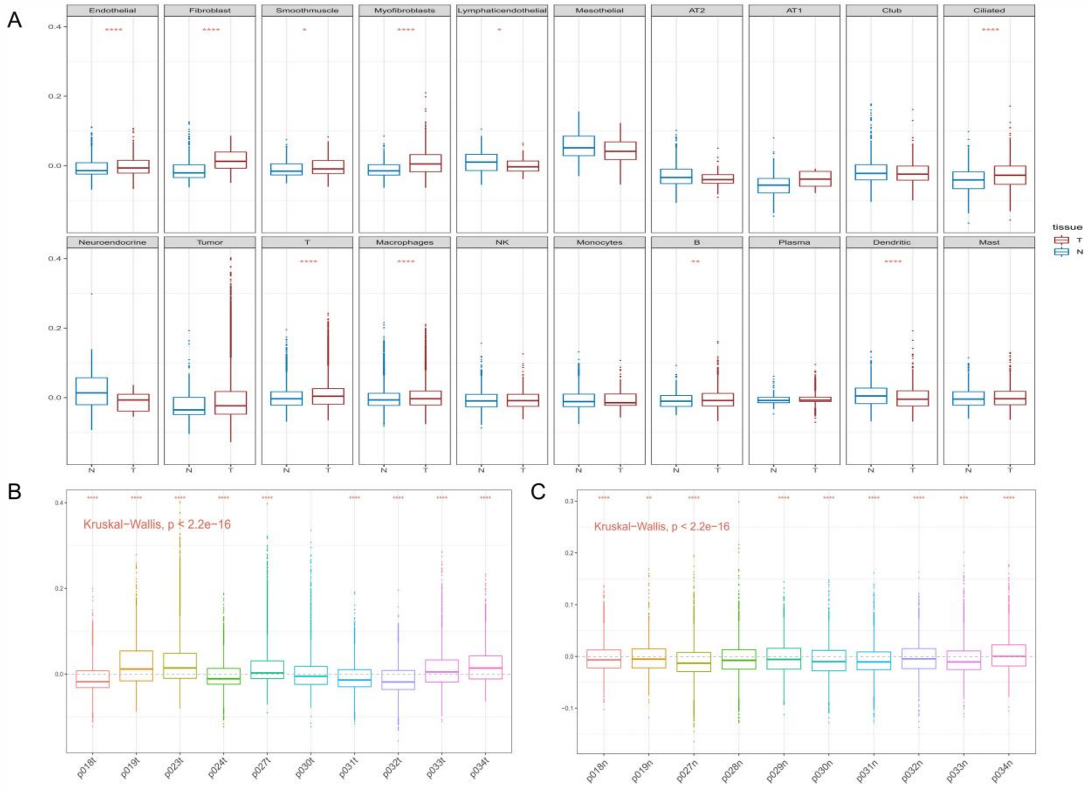


**Figure S4** The CuS difference in samples. **(A)** The difference of CuS in various cell types between normal and tumor samples. **(B)** The CuS in tumor samples. **(C)** The CuS in normal samples.

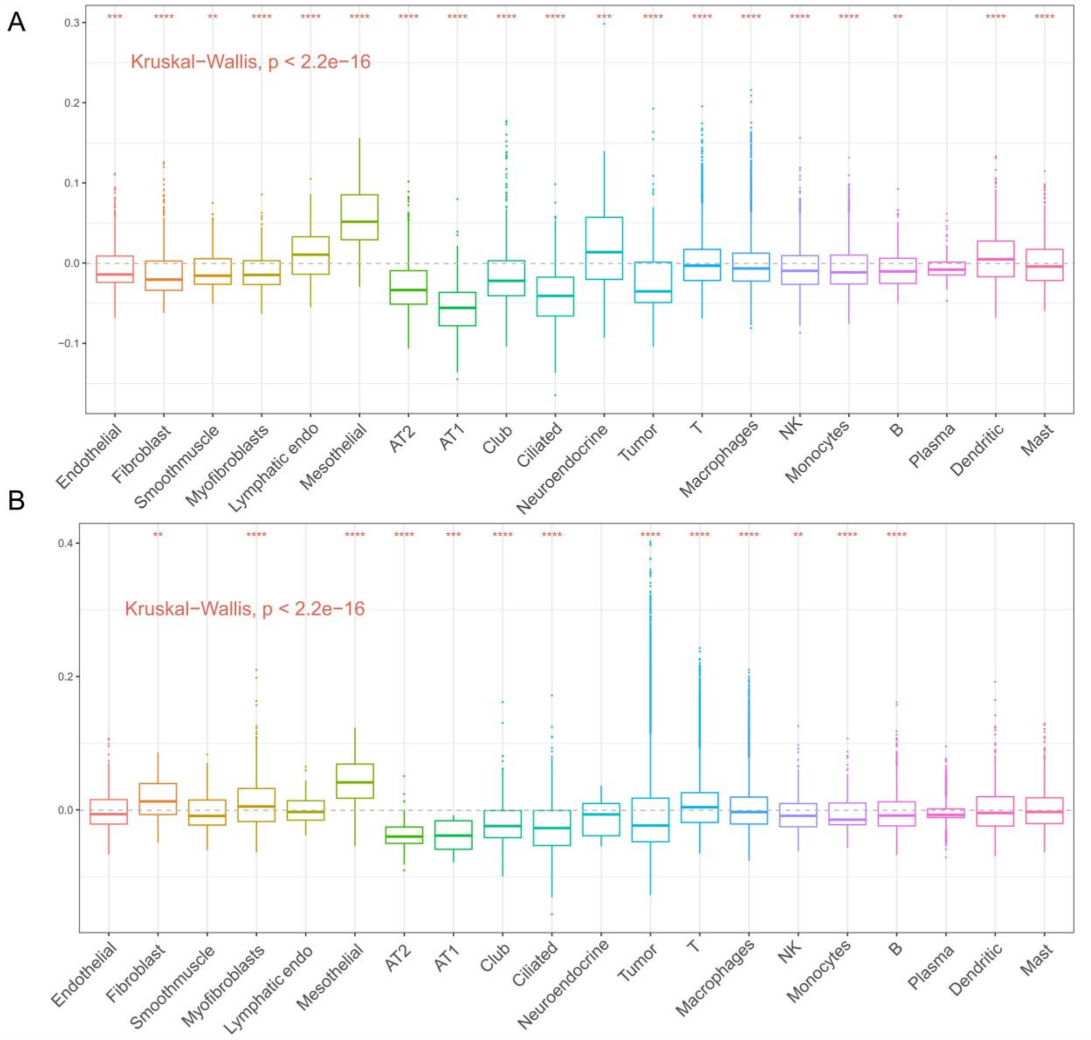


**Figure S5** The CuS in various cells. **(A)** The CuS of various cells in normal samples. **(B)** The CuS of various cells in tumor samples.

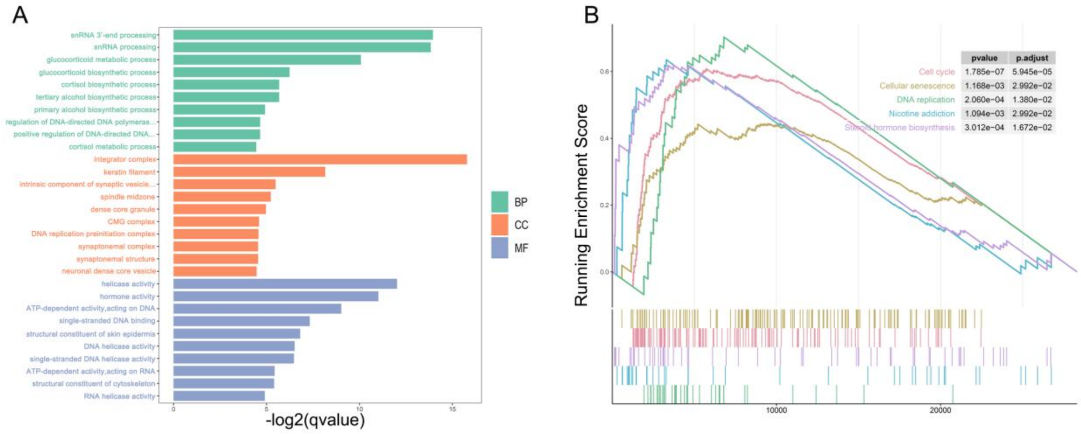


**Figure S6** Function enrichment in TCGA. GO **(A)** and KEGG **(B)** analysis between high and low CuS groups in TCGA data.

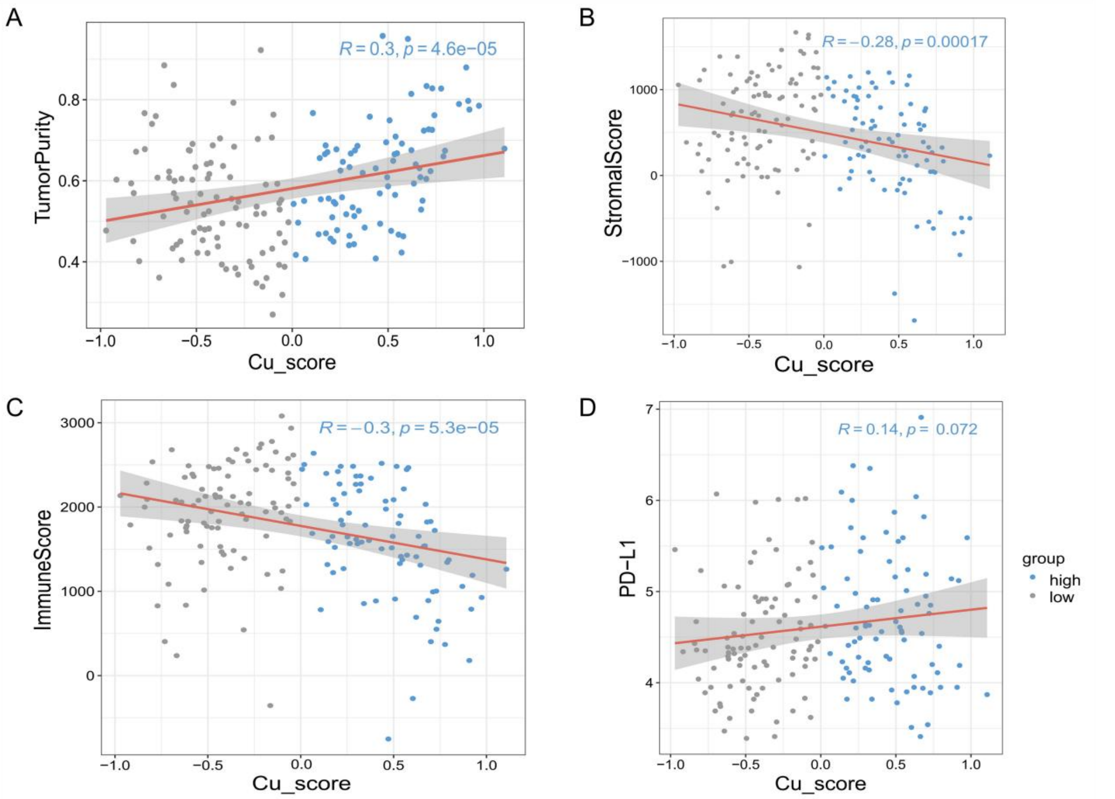


**Figure S7** Immune characteristics of the CuS in GSE42127. **(A)** The CuS was positively associated with tumor purity. **(B)** The CuS was negatively associated with stromal scores. **(C)** The CuS was negatively associated with immune scores. **(D)** The CuS was positively associated with PD-L1 expression.

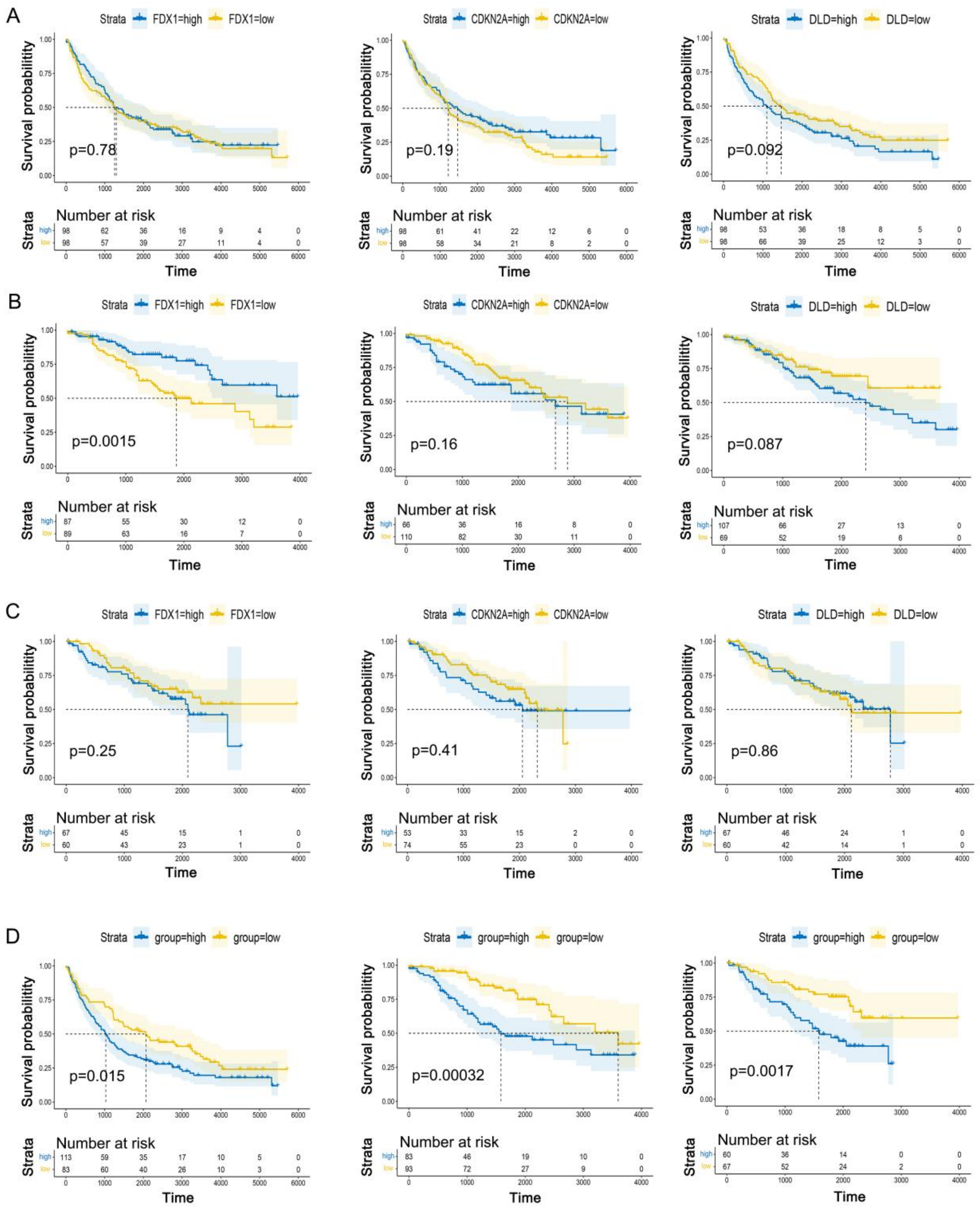


**Figure S8** Comparison between cuproptosis genes and CuS in validation datasets. **(A)** Kaplan-Meier survival analysis of FDX1, DLD and CDKN2A in GSE37745. **(B)** Kaplan-Meier survival analysis of FDX1, DLD and CDKN2A in GSE42127. **(C)** Kaplan-Meier survival analysis of FDX1, DLD and CDKN2A in GSE50081. **(D)** Kaplan-Meier survival analysis of the two CuS groups. Patients with high CuS had significantly worse prognoses than patients with low CuS in the GSE37745(left) , GSE42127(middle) and GSE50081 (right) cohorts.
